# Vitamin D Status, Fasting Blood Glucose, and Latent Tuberculosis Infection in a High-Risk Population in Ulaanbaatar, Mongolia

**DOI:** 10.3390/nu17193122

**Published:** 2025-09-30

**Authors:** Davaasambuu Ganmaa, Sukhbaatar Ariunbuyan, Polyna Khudyakov, Enkhtsetseg Tserenkhuu, Sunjidmaa Bolormaa, Buyanjargal Uyanga, Batbayar Ochirbat, Erkhembulgan Purevdorj, J. Lucian Davis

**Affiliations:** 1 Channing Division of Network Medicine, Brigham and Women’s Hospital, Harvard Medical School, Boston, MA 02115, USA; 2 Department of Nutrition, Harvard T.H. Chan School of Public Health, Boston, MA 02115, USA; 3 Division of Oral and Maxillofacial Oncology and Surgical Sciences, Graduate School of Dentistry, Tohoku University, Sendai 980-8575, Japan; 4 Laboratory of Biomedical Engineering for Cancer, Graduate School of Biomedical Engineering, Tohoku University, Sendai 980-8575, Japan; 5 Sage Therapeutics, Cambridge, MA 02142, USA; 6 Mongolian Health Initiative, Ulaanbaatar 14210, Mongolia; 7 National Center for Communicable Disease, Ulaanbaatar 13335, Mongolia; 8 Ulaanbaatar City Health Department, Ulaanbaatar 14210, Mongolia; 9 Pulmonary, Critical Care, and Sleep Medicine Section, Department of Medicine, Yale School of Medicine, New Haven, CT 06510, USA

**Keywords:** tuberculosis (TB) infection, TB household contacts, healthcare workers, fasting blood glucose, vitamin D deficiency, overweight, obesity, metabolic disease, Prime Diet Quality Score, Mongolia

## Abstract

**Background:** Mongolia is experiencing a rapid epidemiologic transition in which high burdens of micronutrient malnutrition, infection, and cardiometabolic disease are simultaneously prevalent. This cross-sectional study sought to understand how nutritional, lifestyle, and cardiometabolic risk factors are distributed among a population at high-risk for tuberculosis (TB), comprising household contacts (HHCs) and healthcare workers, (HCWs) in Ulaanbaatar, Mongolia, and how these factors are associated with TB infection. **Methods:** A total of 196 HHCs and 241 HCWs were assessed for latent TB infection (LTBI) using the QuantiFERON-TB Gold Plus (QFT-Plus) assay and for diabetes using fingerprick samples for fasting blood glucose. Participants also underwent assessments of their diet and physical activity, nicotine dependence, body mass index, and serum 25(OH)D concentration. We examined associations between assessed risk factors and LTBI using multivariate logistic regression. **Results:** The prevalence of LTBI was 47% for both HHCs and HCWs. A total of 54% percent of HHCs and 68% of HCWs had low physical activity levels; 63% of HHCs and 95% of HCWs were overweight or obese; 7% of HHCs and 4% of HCWs had impaired or diabetic fasting blood glucose [FBG]; and 49% of HHCs and 70% of HCWs were vitamin D deficient. In a multivariable analysis of HHCs, LTBI was independently associated with lower serum [25(OH)D], and the odds ratio (OR) was 3.18 (95% CI 1.38–7.79; *p* = 0.009). In contrast, the probability of LTBI did not differ significantly between vitamin D-deficient and non-deficient HCWs, and the OR was 0.89 (95% CI 0.59–1.37; *p* = 0.42). In a pooled analysis of HHCs and HCWs, the probability of LTBI did not significantly differ between vitamin D-deficient vs. non-deficient participants. The association between serum [25(OH)D] and LTBI among HHCs and HCWs was significantly modified by fasting blood glucose (FBG), such that a lower vitamin D status was significantly more common among those in the highest tertile of FBG than among those in the lowest tertile of FBG. **Conclusions:** Nutritional, lifestyle, and cardiometabolic risk factors are highly prevalent among HHCs and HCWs with TB in Ulaanbaatar, Mongolia. These findings underscore the importance of simultaneously controlling TB infection, malnutrition, and cardiometabolic risks among HHCs and HCWs to reduce the disease burden in Mongolia.

## 1. Background

Mongolia is a rapidly developing lower middle-income country with the world’s highest annual incidence of cancer deaths [1]. It offers a highly relevant setting to investigate the double burden of non-communicable and infectious disease risk factors. Since 1990, Mongolia has seen improvements in a wide variety of heath indicators, from childhood stunting (decreased from 33.1 to 6.1%), under age five mortality (decreased from 103.2 to 17.2 per 1000 persons), life expectancy (increased from 60.5 to 69.5 years), and estimated disability-adjusted life years (DALYs) attributable to respiratory tract infections (−89%), tuberculosis (TB) (−66%), and dietary iron deficiency (−49%) [2,3,4]. Despite these improvements, Mongolia’s TB burden remains among the highest TB burden countries in the Western Pacific and is 10th globally at 428 per 100,000 per year [5,6,7,8]. Some 21.4% of pregnant women remain anemic [3], and dietary and biochemical deficiencies of multiple micronutrients persist among women and children [9,10,11,12,13]. Previously, we reported 25(OH)D levels in Ulaanbaatar women of reproductive age in March–April to be the lowest of any study population globally (79.3% were <10 ng/mL) [14]. Moreover, rising DALYs attributable to smoking (+27%), hypertension (+16%), elevated body mass index (+64%), fasting blood glucose (+19%), and LDL cholesterol (+10%) mean that ischemic heart disease, stroke, and other cardiovascular diseases (CVDs) now kill ~37% of Mongolians [4]. An unhealthy diet is the primary underlying contributor to cardiometabolic risk in Mongolia, to which 63% of male and 61% of female age-standardized CVD deaths are attributed (the highest national percentages for both sexes globally). Mongolia offers a highly relevant setting to develop and evaluate a screening intervention among household contacts to reduce the risk of non-communicable diseases (NCDs), as well as TB, given the high prevalence of obesity (50%), unhealthy diets (30%), smoking (29%), and unhealthy alcohol use (28%).

Therefore, we sought to determine the frequency of these and other TB risk factors and their association with LTBI among Mongolian household TB contacts (HHCs) and healthcare workers (HCWs) as a part of our Zero TB Ulaanbaatar Initiative. Healthcare workers are one of the vulnerable groups to TB infection due to occupational exposure from patients [15,16]. Previous surveys of LTBI among doctors and nurses in Mongolia reported a significant relationship between the tuberculin skin test (TST) and workplaces, with 84.3% of HCWs providing TB services vs. 49.2% HCWs who were not involved in TB services [17]. In this study, we used the QuantiFERON-TB Gold Plus (QFT-Plus) assay, the most sensitive and specific assay for TB infection in a population like Mongolia, where BCG vaccination at birth is nearly universal with booster shots later in life, and focused on Ulaanbaatar, where 50% of Mongolians live and where the majority of TB occurs in the country [7].

## 2. Methods

### 2.1. Study Population

In this cross-sectional study, we assessed household contacts (HHCs) of TB patients and healthcare workers in Ulaanbaatar, Mongolia. Household contacts were defined as individuals living in the same household or within the same fenced compound as a newly diagnosed TB patient during the three months prior to diagnosis. Newly diagnosed TB patients themselves were not included as HHC participants. Instead, healthcare workers interviewed these patients to identify their household members, who were then invited by phone to attend informational meetings about the study at district TB dispensary training centers. Contacts with known active TB disease, those already receiving TB treatment, or the immunosuppressed were excluded. Meetings were held at the training centers of district TB dispensaries. Healthcare workers were identified as eligible by their hospital’s administration and invited by phone to attend informational meetings held at the training centers of district TB dispensaries or the National Center for Communicable Disease (NCCD).

### 2.2. Measurements

Participant height was measured to the nearest 0.1 cm using a portable stadiometer (seca GmbH & Co., Hamburg, Germany). Weight was then measured to the nearest 0.1 kg using a Digital Floor Scale (seca GmbH & Co.). Body mass index (BMI) was calculated using height and weight and categorized according to Asian-specific cutoffs [18,19].

### 2.3. Questionnaire Data

We piloted a set of screening instruments to assess potential lifestyle risk factors for TB infection and adjust for potential confounders in other associational analyses: an instrument for collecting the Prime Diet Quality Score (PDQS) and three internationally validated instruments—the Pittsburgh Sleep Quality Index (PSQI), International Physical Activity Questionnaire Short Form (IPAQ), and the Fagerstrom Test for Nicotine Dependence [20,21,22,23,24]. IPAQ data were analyzed using an Excel system developed by Cheng [25] to track total minutes and days per week of physical activity, MET (metabolic equivalent) minutes per week, and physical activity category. Total energy expenditure (TEE) was also estimated by summing the calorie equivalent of calculated METs and basal metabolic rate calculated using age- and sex-specific equations based on height and weight [26]. We condensed Fagerstrom nicotine dependence scores in categorical terms according to published guidance [27]. In analyzing the PDQS, increasing consumption frequency of positively scored components (<1/week, 1/week, 2–4/week, 5–7/week, 2+/day) were given increasing subscores (0, 1, 2, 3, 4), respectively, increasing frequency of negatively scored components were given decreasing subscores (4, 3, 2, 1, 0). All components’ subscores were summed to produce a total score for use in regression models. Three sets of regression models were fitted:

- Model 1 (age- and sex-adjusted): Adjusted for age and sex. Age was modeled using natural splines with two degrees of freedom.
- Model 2 (multivariable-adjusted): Further adjusted for smoking status, Prime Diet Quality Score, and minutes per week of physical activity.
- Model 3 (multivariable-adjusted + BMI): Additionally adjusted for body mass index (BMI).

### 2.4. Biochemical and Anthropometric Data

Fingerprick samples were collected from each participant by trained research fellows after ascertaining their fasting state using a GluNEO^®^ Lite test glucometer (Infopia Ltd., Anyang, Republic of Korea). For detecting latent TB infection (LTBI), a peripheral blood sample was drawn in high-altitude tubes for a QuantiFERON-TB Gold Plus (QFT-Plus) (Qiagen, Hilden, Germany) assay. After incubation for 16 hours, serum was separated, and the interferon-gamma release assay (IGRA) was performed in the Global Laboratory (Ulaanbaatar, Mongolia) according to the manufacturer’s instructions. Another set of serum samples was frozen at −80 °C until analyzed for total serum 25(OH)D concentration determined by the VIDAS^®^ enzyme-linked fluorescent assay (ELFA) (Biomérieux, Marcy-L’Etoile, France) and was analytically certified by participation in the DEQAS (Vitamin D External Quality Assessment Scheme) program [28].

### 2.5. Statistical Analysis

The descriptive results of the questionnaires were tabulated. All study variables were described as categorical and summarized using frequencies and percentages, including age group, sex, PSQI score, IPAQ physical activity level, self-reported sleep quality, smoking status, BMI category, QFT-Plus results, vitamin D (25(OH)D) status, and venous blood glucose level. Results were presented in tabular form. Associations between demographic, lifestyle, and clinical factors and latent TB infection (outcome status, yes/no) were evaluated using unadjusted and multivariable logistic regression models, ran both pooled and separately for HHCs and HCWs. Logistic regression was chosen because it models the odds of infection directly; in strata where outcomes were relatively uncommon, odds ratios approximate prevalence ratios. To aid interpretability, model-based predicted probabilities with 95% confidence intervals are presented alongside odds ratios. Vitamin D status was categorized as deficient (<20 ng/mL) or non-deficient (≥20 ng/mL). Fasting blood glucose (FBG) was classified according to WHO guidelines: normal (<6.1 mmol/L), impaired (6.1–7.0 mmol/L), and diabetic (≥7 mmol/L) [29]. To enhance statistical power, pooled analyses of HHCs and HCWs were also performed to evaluate potential effect modification between vitamin D status and FBG using interaction terms. In sensitivity analyses, smaller glucose categories (<4.6, 4.6–5.1, >5.1 mmol/L) were also applied. To evaluate potential effect modification between vitamin D status and fasting blood glucose (FBG), interaction terms were included in logistic regression models. From these models, we derived model-based predicted probabilities of latent TB infection for each subgroup. Differences (contrasts) in predicted probabilities between vitamin D-deficient and non-deficient groups were reported to illustrate the magnitude of effect modification, as these provide a more intuitive interpretation than ratios of odds ratios. Corresponding *p*-values for the interaction terms were also reported.

Consumption frequencies of food groups among participants were graphed by participant type (household contacts and healthcare workers). Principal component analysis (PCA) was used as a descriptive method to identify dietary patterns among participants, stratified by participant type (household contacts and healthcare workers). Consumption frequencies of food groups were converted into point values (<1/week: 1 point; 1/week: 2 points; 2–4/week: 3 points; 5–6/week: 4 points; ≥1/day: 5 points). Patterns were retained based on inspection of the scree plot and other quantitative and qualitative criteria [30]. Factor loadings were used to calculate pattern scores for each participant (scaled 0–100), and mean adherence to each factor was computed by participant type and sex. All analyses were performed in R v. 3.5.3 using the “prcomp” package.

## 3. Results

Table 1 describes tabulated descriptive statistics for HHCs, HCWs, and the pooled population. HCWs were older (mean age: 42.0 ± 9.9 years) and predominantly female (80%), while HHCs were younger (mean age: 31.5 ± 18.1 years), and 50% were female. Vitamin D deficiency (<20 ng/mL) was present in 71% of HCWs and 50% of HHCs. Among HHCs, 7% had impaired (5%) or diabetic (2%) FBG (≥6.1 mmol/L), whereas 4% of HCWs had impaired (3%) or diabetic (1%) FBG. The prevalence of latent TB infection was 47% in both populations. As measured by the IPAQ, 48% of HCWs and 54% of HHCs had low levels of physical activity in the past month. As measured by the PSQI, 48% of HCWs and 21% of HHCs had fairly poor or very poor sleep quality in the past week. Only two HCWs (<1%) reported smoking as compared to 19% of HHCs, and 95% of HCWs and 63% of HHCs were overweight or obese.

The age- and sex-adjusted probability of LTBI among vitamin D-deficient HHCs was 59% (95% CI 43–73%), and among non-deficient HHCs it was 36% (95% CI 20–56%) (*p* = 0.018), with an OR of 2.55 (95% CI 1.19–5.65) (Model 1, Table 2). This association was also significant in a model with an additional adjustment for the smoking status, Prime Diet Quality Score, and minutes per week of physical activity, with an OR of 2.77 (95% CI 1.26–6.37) (*p* = 0.013) (Model 2, Table 2), and in a multivariate model with a further adjustment for the BMI, with an OR of 3.18 (95% CI 1.38–7.79) (*p* = 0.009) (Model 3, Table 2).

Vitamin D status was not associated with LTBI among HCWs or the overall population (Table 2). Odd ratios for the combined population were 1.09 (95% CI, 0.78–1.53) in the age- and sex-adjusted model; 1.09 (95% CI, 0.79–1.53) in a model with additional adjustments for smoking status, the Prime Diet Quality Score, and minutes per week of physical activity; and 1.08 (95% CI, 0.78–1.53 in a multivariate model with a further adjustment for the BMI (*p* = 0.49).

A significant interaction between the FBG and vitamin D status was observed (Table 3) in the combined population. In multivariable-adjusted models, QFT+ probability decreased from 56% to 35%, with increasing tertiles of FBG among those with 25(OH)D ≥ 20 ng/mL. It increased from 44% to 60% among those with 25(OH)D < 20 ng/mL (p for interaction = 0.012). A significant interaction was also found in the age- and sex-adjusted model (*p* = 0.012) and the multivariable model further adjusted for BMI (*p* = 0.008). Within the highest tertile of FBG (>5.1 mmol/L), there was a significant multivariable-adjusted contrast in the probability of QFT+ among those with 25(OH)D < 20 ng/mL (60%) and 25(OH)D ≥ 20 ng/mL (35%) (*p* = 0.018) (Table 3); significant contrasts were also found in the age- and sex-adjusted model (*p* = 0.017) and multivariable model further adjusted for BMI (*p* = 0.013). No significant contrast was observed between 25(OH)D < 20 and ≥20 ng/mL groups among those with a FBG < 4.6 or 4.6–5.1 mmol/L in any models. Because the BMI distribution was highly skewed among HCWs (very few normal-weight participants), we did not evaluate BMI interactions a priori; instead, the BMI was included as an adjustment variable (Model 3). We note this as a limitation.

### Diet Patterns

The diets among healthcare workers (HCWs) were generally more diverse in that most food groups were consumed in greater frequencies than in household contacts (HHCs). Three diet patterns were observed (Table S1): an “urban traditional” pattern (attributing 29% of the variance in intake of factor components), marked by an exceptionally high consumption of red meat and a low consumption of fruits, vegetables, lean meats (poultry and fish), and all other components; an “urban modern” pattern (attributing 14% of variance), marked by a high consumption of lean meats and a low consumption of red meat, refined grains, milk and dairy, and potatoes; and an “urban transitional” pattern, marked by a high consumption of some “traditional” components, such as refined grains and potatoes, as well as some “modern” ones, including sugary drinks and fried foods. The adherence to the urban traditional pattern was higher among household contacts (HHCs), and adherence to the urban modern pattern was higher in healthcare workers (HCWs) (Table S2). Sex differences in the pattern adherence within participant types were less salient than differences between participant types (Table S3).

## 4. Discussion

Many clinical, behavioral, socioeconomic, and environmental variables affect the risk of TB infection and the progression of latent TB infection to active TB disease, including malnutrition, air pollution, HIV and other co-infections, smoking and alcohol use, diabetes, and poverty [6,31]. The health and well-being of TB household contacts and healthcare workers are a significant public health concern, as they affect the burden of TB among them and the larger population. In this study of a population with a high-risk of TB in Ulaanbaatar, Mongolia, we found a high prevalence of TB infection in terms of dietary risks, physical inactivity, nicotine dependence, overweight status and obesity, impaired glucose metabolism, and vitamin D deficiency.

Prior studies have suggested that vitamin D deficiency increases the risk of TB infection and the progression of latent to active TB [32] and may play a role in the increased risk of TB observed among people with diabetes [33,34]. In an observational analysis of 9810 Ulaanbaatar schoolchildren, we found vitamin D deficiency to be a significant independent risk factor for TB infection [28]. In the current study, we observed similar associations between vitamin D and latent TB infection in a population of TB household contacts but not in healthcare workers. The association between vitamin D and TB was significantly modified by fasting blood glucose, such that vitamin D appeared most protective against TB infection among those with higher tertiles of fasting blood glucose levels. These findings are particularly significant in the context of Mongolia, a high TB burden country [6,7] where 10% of adults have diabetes [35] and 47% of males smoke (ranking 25th among all countries) [2] and where we previously found 42% of adults nationwide to have 25(OH)D levels below 20 ng/mL in summer and 99% in winter [30,36]. Differences between HHCs and HCWs may reflect higher and more heterogeneous occupational exposure among HCWs, potentially overwhelming modest host factor effects such as vitamin D status. Although, models adjusted for the BMI, residual confounding by unmeasured occupational factors, and limited power within strata may explain the lack of association among HCWs. Given this context, the marginal benefit of smoking cessation and vitamin D supplementation, fortification, and other remediation strategies on TB risk in the Mongolian population could be substantial and warrants clinical research to confirm this. In Mongolia, dietary vitamin D mainly comes from animal-based foods, such as fortified milk, liver, dairy products, and eggs.

Despite the well-established association between diabetes mellitus (DM) and active tuberculosis (TB) [37], it is still not clear whether persons with impaired glucose metabolism have a higher risk for LTBI [38]. Although vitamin D deficiency might be a risk factor for LTBI [39], there is no direct evidence suggesting an association between impaired glucose metabolism and vitamin D deficiency in individuals with LTBI except for one study that evaluated the association between insulin resistance and vitamin D deficiency in individuals with LTBI based on the NHANES data [40]. Our study observed a significant interaction between FBG and vitamin D status in predicting latent TB infection, but the causal relationship between fasting blood glucose and vitamin D in individuals with LTBI could not be inferred due to the cross-sectional study design. Also, a residual bias from unmeasured confounders or missing data might affect the results of this study. Specifically, the data was incomplete for the PSQI scores, self-reported sleep quality, BMI, and 25(OH)D concentrations. Having complete data on these variables could have enhanced the model adjustment and lessened the bias, underscoring the need for the systematic collection of such covariates in future research. The observed association should be confirmed in a more extensive national survey including more individuals with impaired glucose metabolism or diabetes. Despite these limitations, the current study has described a high prevalence of modifiable risk factors for TB in Mongolia, including diet, low physical activity, smoking, and obesity, reinforcing the importance of concurrently addressing non-communicable and infectious disease risk factors in Mongolia [2,41], particularly among high-risk groups.

## 5. Conclusions

This study describes the endemic double burden of under- and over-nutrition and chronic and infectious diseases among the high-risk for TB population, namely household contacts and healthcare workers in Ulaanbaatar, Mongolia. Public health programs should prioritize the mitigation of nutritional, lifestyle, and cardiometabolic risks among this high-risk group—including smoking cessation, weight reduction, physical exercise, vitamin D supplementation, and dietary modification—as part of national efforts to control infectious and chronic diseases. Research is also warranted to better understand the relationship between TB, vitamin D, and fasting blood glucose and the implications of this interaction for diabetics and prediabetics living in high TB burden communities globally.

## Figures and Tables

**Table 1:** Characteristics of study population.

Characteristic		HHC		HCW		Overall	
		*n*/Total	%	*n*/Total	%	*n*/Total	%
Age (years)	<20	66/193	34	0/240	0	66/434	15
	20 to 29	26/193	14	28/240	12	54/434	12
	30 to 39	33/193	17	83/240	35	116/434	27
	40 to 49	30/193	16	66/240	28	96/434	22
	50 to 59	26/193	14	58/240	24	85/434	20
	60+	12/193	6	5/240	2	17/434	4
Female sex		103/195	1	199/241	1	303/437	1
IPAQ physical activity category	Low	106/195	54	162/238	68	269/434	62
	Medium	57/195	29	57/238	24	114/434	26
	High	32/195	16	19/238	8	51/434	12
PSQI score	fine	144/183	79	118/230	51	262/414	63
	Poor (>5)	39/183	21	111/230	48	151/414	37
Self-reported sleep quality	Very good	79/195	41	1/241	0	80/437	18
	Fairly good	94/195	48	41/241	17	135/437	31
	Fairly poor	20/195	10	121/241	50	141/437	32
	Very poor	0/195	0	66/241	27	67/437	15
Smoker		37/195	19	2/240	1	39/436	9
BMI	Underweight (<18.5)	1/195	1	0/241	0	1/437	0
	Normal (18.5–24.9)	66/195	34	7/241	3	73/437	17
	Overweight (25–29.9)	103/195	53	174/241	72	278/437	64
	Obese (>30)	20/195	10	55/241	23	75/437	17
QFT-Plus positive		93/195	48	110/241	46	203/437	47
25(OH)D	Deficient (<10 ng/mL)	24/195	12	120/241	50	144/437	33
	Insufficient (10–20 ng/mL)	73/195	37	52/241	22	126/437	29
	Adequate (20–30 ng/mL)	39/195	20	31/241	13	70/437	16
	Optimal (30+ ng/mL)	8/195	4	31/241	13	39/437	9
Venous blood glucose	Normal (<6.1 mmol/L)	182/195	93	205/213	96	388/409	95
	Impaired (6.1 to 7 mmol/L)	9/195	5	6/213	3	15/409	4
	Diabetic (>7 mmol/L)	4/195	2	2/213	1	6/409	1.5

**Footnote**: Please note that for some characteristics the % does not sum up to 100% because of missing data. Abbreviations: QFT-Plus Positive, QuantiFERON-TB Gold Plus Positive; 25(OH)D, 25-hydroxyvitamin D; IPAQ, International Physical Activity Questionnaire; PSQI, Pittsburgh Sleep Quality Score; and BMI; Body Mass Index.

**Table 2:** Estimated probability of latent TB infection by 25(OH)D among pooled cohort, HHC only and HCW only.

Model	Cohort	Category	P(QFT-Plus) * (95% CI)	OR (95% CI)	*p*-Value
1. Age- and sex-adjusted	Pooled	≥20 ng/mL	0.44 (0.33, 0.56)	Ref	0.483
		<20 ng/mL	0.48 (0.41, 0.56)	1.09 (0.78, 1.53)	
	HHC only	≥20 ng/mL	0.36 (0.20, 0.56)	Ref	0.018
		<20 ng/mL	0.59 (0.43, 0.73)	2.55 (1.19, 5.65)	
	HWC only	≥20 ng/mL	0.54 (0.38, 0.69)	Ref	0.454
		<20 ng/mL	0.48 (0.38, 0.59)	0.90 (0.6, 1.38)	
2. Multivariable-adjusted	Pooled	≥20 ng/mL	0.44 (0.33, 0.56)	Ref	0.475
		<20 ng/mL	0.48 (0.40, 0.56)	1.09 (0.79, 1.53)	
	HHC only	≥20 ng/mL	0.35 (0.18, 0.56)	Ref	0.013
		<20 ng/mL	0.60 (0.43, 0.75)	2.77 (1.26, 6.37)	
	HWC only	≥20 ng/mL	0.53 (0.37, 0.69)	Ref	0.467
			0.47 (0.37, 0.58)	0.90 (0.6, 1.39)	
3. Multivariable-adjusted + BMI	Pooled	≥20 ng/mL	0.46 (0.35, 0.59)	Ref	0.486
		<20 ng/mL	0.51 (0.42, 0.59)	1.08 (0.78, 1.53)	
	HHC only	≥20 ng/mL	0.43 (0.23, 0.65)	Ref	0.009
		<20 ng/mL	0.70 (0.52, 0.84)	3.18 (1.38, 7.79)	
	HWC only	≥20 ng/mL	0.54 (0.38, 0.70)	Ref	0.416
		<20 ng/mL	0.48 (0.37, 0.59)	0.89 (0.59, 1.37)	

**Footnote**: Abbreviations: QFT-Plus Positivity, QuantiFERON-TB Gold Plus Positivity; and 25(OH)D, 25-hydroxyvitamin D. Probabilities are based on the model QFT-Plus result = 25(OH)D category + Model 1: age (age-adjusted models), gender (male/female); Model 2: age (age-adjusted models), gender (male/female), smoking status, Prime Diet Quality Score, and minutes per week of physical activity; and Model 3: age (age-adjusted models), gender (male/female), smoking status, Prime Diet Quality Score, and minutes per week of physical activity and body mass index. Age is modeled using natural splines with two degrees of freedom. * Probability of QFT-Plus positivity.

**Table 3:** Interaction between serum 25(OH)D and fasting blood glucose in predicting latent TB infection among a pooled cohort.

	25(OH)D	FBG < 4.6 mmol/L			FBG 4.6–5.1 mmol/L			FBG ≥ 5.1 mmol/L			*p* Value **
		P(QFT-Plus) *	95% CI		P(QFT-Plus) *	95% CI		P(QFT-Plus) *	95% CI		
Model 1	<20 ng/mL	44%	34%	55%	46%	33%	59%	61%	45%	75%	0.012
	≥20 ng/mL	56%	38%	72%	45%	28%	64%	37%	20%	57%	
P for contrast (<20 vs. ≥20 ng/mL)		0.254			0.948			0.017			
Model 2	<20 ng/mL	44%	33%	55%	45%	32%	58%	60%	44%	74%	0.012
	≥20 ng/mL	56%	38%	72%	43%	26%	62%	35%	19%	56%	
P for contrast (<20 vs. ≥20 ng/mL)		0.246			0.887			0.018			
Model 3	<20 ng/mL	45%	34%	57%	50%	36%	64%	66%	50%	80%	0.008
	≥20 ng/mL	58%	40%	75%	50%	31%	69%	39%	21%	61%	
P for contrast (<20 vs. ≥20 ng/mL)		0.214			0.996			0.013			

**Footnote**: Abbreviations: P, Probability; QFT-Plus Positivity, QuantiFERON-TB Gold Plus Positivity; 25(OH)D, 25-hydroxyvitamin D; and FBG, Fasting Blood Glucose. Probabilities are based on the model QFT-Plus result = 25(OH)D category or FBG category + Model 1: age (age-adjusted models), gender (male/female); Model 2: age (age-adjusted models), gender (male/female), smoking status, Prime Diet Quality Score, and minutes per week of physical activity; and Model 3: age (age-adjusted models), gender (male/female), smoking status, Prime Diet Quality Score, and minutes per week of physical activity and body mass index. Age is modeled using natural splines with two degrees of freedom; * Probability of QFT-Plus positivity; ** P for interaction between 25(OHD)D and low level of FBG.

## Data Availability

The original contributions presented in the study are included in the article/Supplementary Materials, further inquiries can be directed to the corresponding author.

## Supplementary Materials

The following supporting information can be downloaded at: https://www.mdpi.com/article/10.3390/nu17193122/s1, Table S1: Diet patterns factor loadings; Table S2: Mean diet pattern scores by participant group; Table S3: Mean diet pattern scores by participant group and sex.
