# Supplementary material for: Vitamin D Status, Fasting Blood Glucose, and Latent Tuberculosis Infection in a High-Risk Population in Ulaanbaatar, Mongolia
Source: Nutrients. 2025 Sep 30;17(19):3122. doi: 10.3390/nu17193122 (PMC12525777; doi:10.3390/nu17193122)
Supplement: Supplementary file 1 [file nutrients-17-03122-s001.zip › nutrients-3879974-supplementary.pdf]

Supplementary Materials:

Table S1: Diet patterns factor loadings

Food Group	Urban Traditional	Urban Modern	Urban Transitional
Red Meat	0.324	-0.591	0.004
Refined Grains & Products	-0.022	-0.654	0.272
Liquid Oils	-0.049	-0.639	-0.171
Milk & Milk Products	-0.117	-0.591	0.051
Potatoes & Other White Tubers	-0.131	-0.537	0.312
Sweets & Ice Cream	-0.266	-0.368	0.532
Whole Grains & Products	-0.302	-0.364	-0.462
Other Vegetables	-0.367	-0.298	-0.436
Sugary Drinks	-0.433	-0.290	0.505
Eggs	-0.462	-0.397	-0.102
Fried Foods Outside Home	-0.499	0.108	0.259
Cruciferous Vegetables	-0.548	-0.272	-0.444
Deep Orange Fruits & Vegetables	-0.563	-0.157	-0.019
Dark Green Leafy Vegetables	-0.580	-0.156	-0.472
Citrus Fruits	-0.654	-0.105	0.194
Processed Meat	-0.671	0.222	0.184
Other Fruits	-0.733	0.118	0.146
Fish	-0.781	0.305	0.052
Nuts & Seeds	-0.782	0.134	-0.082
Poultry	-0.788	0.251	0.056
Legumes	-0.802	0.232	-0.046

Footnote: Urban Traditional, Urban Modern, and Urban Transitional diet patterns account for 28.3%, 13.6%, and 8.3% of variation in intake of pattern components, respectively (total: 50.2%). Factor loadings were generated using dietary intake for all participants 5 years and older after assigning point values to reported frequencies as follow (<1/wk: 1 point, 1/wk: 2 points, 2-4/wk: 3 points, 5-6/wk: 4 points, 1+/day: 5 points).

Table S2: Mean diet pattern scores by participant group

Pattern	Household contacts	Healthcare Workers
Urban Traditional	29.2	-18.0
Urban Modern	-6.6	4.0
Urban Transitional	0.9	-0.6

Footnote: Means are calculated among participants 5 years and older. Pattern scores are scaled from 0–100.

Table S3: Mean diet pattern scores by participant group and sex

	**Household contacts**		**Healthcare Workers**	
	**Male**	**Female**	**Male**	**Female**
Urban Traditional	30.7	28.0	-29.0	-15.7
Urban Modern	-6.6	-6.6	4.8	3.9
Urban Transitional	3.2	-1.0	0.0	-0.7

Footnote: Means are calculated among participants 5 years and older. Pattern scores are scaled from 0–100.
